# Efficacy assessments of SF001, a next-generation polyene antifungal, in a neutropenic mouse model of invasive fusariosis

**DOI:** 10.1128/aac.01802-24

**Published:** 2025-04-01

**Authors:** Teclegiorgis Gebremariam, Yiyou Gu, Hoja Patterson, Eman Youssef, Sondus Alkhazraji, Tasneem Elsayed, Nathan P. Wiederhold, Ashraf S. Ibrahim

**Affiliations:** 1 Division of Infectious Diseases, The Lundquist Institute for Biomedical Innovation at Harbor-University of California Los Angeles (UCLA) Medical Center 117316 , Torrance, California, USA; 2 The University of Texas Health Science Center at San Antonio 14742 , San Antonio, Texas, USA; 3 David Geffen School of Medicine at UCLA 12222 , Los Angeles, California, USA; University Children's Hospital Münster, Münster, Germany

**Keywords:** SF001, antifungal agents, *Fusarium*, mouse, infection model, liposomal amphotericin B

## Abstract

Fusariosis has high mortality rates with limited treatment options. Owing to its rarity, comparative clinical trials are hard to perform. SF001 is a novel, next-generation polyene drug, rationally designed to reduce potential for systemic toxicity, with long-acting, potent, broad-spectrum fungicidal activity. We compared the *in vitro* activity and *in vivo* efficacy of SF001 with liposomal amphotericin B (LAMB) in treating immunosuppressed mice infected with hematogenously disseminated fusariosis. The minimum inhibitory concentration (MIC) of SF001 and LAMB against *Fusarium solani* or *Fusarium oxysporum* strains (at 100% inhibition) ranged between 0.5–8 µg/mL and 1–>16 µg/mL, respectively. In the hematogenously disseminated fusariosis model, treatment with SF001 or LAMB enhanced the median survival time vs placebo (7, 10, and 9 days at 3, 7.5, and 30 mg/kg of SF001, respectively, and 12.5 days for LAMB at 7.5 mg/kg vs 6.5 days for placebo, *P* < 0.0001). SF001 and LAMB treatment enhanced the overall survival by day 21 (40% and 25% for SF001 at 7.5 mg/kg and 30 mg/kg, respectively, 30% for LAMB at 7.5 mg/kg and 0% for placebo). The survival data were mirrored in the kidney and brain fungal burden results with ~2–3 log_10_ reduction in conidial equivalents/gram for either treatment vs placebo. Furthermore, the reduction in tissue fungal burden was corroborated by histopathological data from target organs, showing reduced or no abscesses in SF001- or LAMB-treated mice. Our data show comparable activity of SF001 to LAMB, thereby supporting the continued development of SF001 for the treatment of invasive fusariosis.

## INTRODUCTION

Fusariosis describes a variety of infections that can vary from superficial, locally invasive, to hematogenously disseminated disease (1). The infection is caused by members of at least 20 species among the genus *Fusarium*, of which the most common human pathogens are members of the *Fusarium solani* species complex (2, 3). Indeed, the *F. solani* complex accounts for approximately half of the reported infections, followed by *Fusarium oxysporum*, *Fusarium moniliforme,* and *Fusarium verticillioides*, each of which account for 10–14% of the total incidence of infections (1, 4–6). *Fusarium* species are a common cause of fungal keratitis and onychomycosis among immunocompetent hosts (1). Other infections in the immunocompetent host have been described and include sinusitis (7), pneumonia (8), endophthalmitis (9), and osteomyelitis (10).

Alarmingly, fusariosis has become increasingly a common problem among patients with hematologic malignancies, particularly in the setting of hematopoietic stem cell transplantation, causing pneumonia, fungemia, and disseminated disease (2, 4). Amphotericin B (AMB) and lipid formulations of amphotericin B, such as liposomal amphotericin B (LAMB), are antifungal agents that are routinely used for treating invasive fusariosis (11), followed by azoles for refractory cases (12). However, AMB and LAMB continue to be associated with considerable toxicities, and azoles have variable activities against different strains. The ineffectiveness of antifungal treatment in patients with hematologic malignancies is highlighted by the high mortality rates of ~70% and almost 100% in patients with persistent neutropenia and disseminated disease (4, 13, 14). Therefore, new antifungal drugs are needed for treating invasive fusariosis.

SF001 (previously, AM-2–19_DP2K) is a novel, next-generation polyene antifungal drug rationally designed to reduce systemic toxicity while maintaining potency, spectrum, and fungicidal activity (15). SF001 demonstrates a broad-spectrum *in vitro* fungicidal activity against a variety of pathogenic fungi including *Candida*, *Aspergillus*, *Cryptococcus*, *Histoplasma*, *Coccidioides*, *Blastomyces*, and *Talaromyces* species, as well as Mucorales fungi (15). Furthermore, SF001 was reported to have *in vivo* activity against a variety of mouse fungal infections including invasive candidiasis due to *Candida glabrata*; hematogenously disseminated aspergillosis due to *Aspergillus fumigatus*, and *Aspergillus terreus*; invasive pulmonary aspergillosis due to *A. fumigatus*; and invasive mucormycosis due to *Rhizopus delemar* and *Mucor circinelloides* (15, 16). More recently, SF001 was also shown to have *in vivo* activity against cryptic aspergilli that are known to be more resistant to current antifungal drugs including *A. lentulus* and *A. calidoustus* (17). Here, we describe the *in vitro* activity of SF001 against *F. solani* and *F. oxysporum* clinical isolates and compare the *in vivo* efficacy of the drug to LAMB using a well-established neutropenic mouse model of hematogenously disseminated *F. solani* infection.

## RESULTS

### SF001 has more favorable *in vitro* activity against *Fusarium* spp. than LAMB

The *in vitro* activity of SF001 against different clinical isolates of *F. solani* or *F. oxysporum* was compared to the activity of AMB or LAMB using the CLSI M38 broth microdilution method (18). The minimum inhibitory concentrations (MICs) that resulted in 50% and 100% inhibition of the growth of the fungal spores were determined after 48 hours of incubation at 35°C. Using the 100% growth inhibition endpoint, SF001 and LAMB had MIC ranges of 0.5–8.0 µg/mL and 1–>16 µg/mL, respectively, against seven clinical isolates of *F. solani* and six clinical isolates of *F. oxysporum* (Table 1). Using the 50% inhibition endpoint, SF001 MIC values ranged from 0.25 to 2 µg/mL, while those for LAMB ranged from 0.06 to >16 µg/mL. The favorable MIC values obtained with SF001 over LAMB were comparable to values obtained for AMB, for which the 100% inhibition endpoint ranged between 0.5 and 2.0 µg/mL (Table 1).

**TABLE 1:** MIC (µg/mL) values at 50% and 100% growth inhibition^*a*^

*F. solani* strain	MIC (µg/mL) 50% inhibition			MIC (µg/mL) 100% inhibition		
	SF001	AMB	LAMB	SF001	AMB	LAMB
95–2478	0.25	ND	1.0	0.5	2	2
FS1	0.5	ND	2	2	2	>16
FS2	1	ND	0.5	4	1	>16
FS3	2	ND	2	4	2	>16
FS4	2	ND	8	8	2	>16
FS5	0.5	ND	0.06	1	2	2
FS6	0.5	ND	0.25	2	0.5	1
*F. oxysporum* strain						
FO1	0.5	ND	1	2	2	>16
FO2	1	ND	8	4	2	>16
FO3	1	ND	8	2	2	>16
FO4	2	ND	2	4	2	>16
FO5	2	ND	>16	4	1	>16
FO6	2	ND	8	4	1	>16

^*a*^ ND, not determined.

### SF001 has comparable activity to LAMB in protecting neutropenic mice from hematogenously disseminated fusariosis

**Fig 1:**
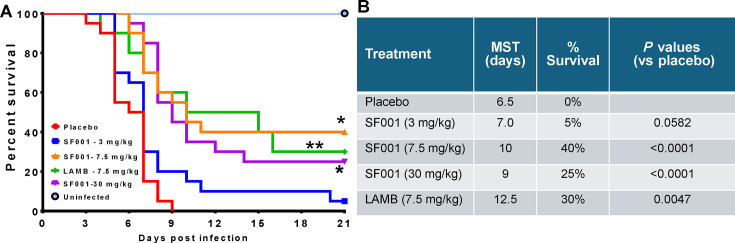
SF001 and LAMB increase survival in immunosuppressed mice infected with *F. solani*. (**A**) Survival of mice infected intravenously with *F. solani* 95–2478 (*n* = 20/group from two independent experiments with average inoculum of 9.1 × 10^2^ spores). *P < 0.01 vs placebo and **P < 0.005 vs placebo and 0.3 mg/kg SF001. (**B**) The table depicts the median survival time (MST) and the overall survival by day 21 post-infection.

To test the activity of SF001 in treating invasive fusariosis, we infected neutropenic mice intravenously with *F. solani* 95–2478 and treated them with SF001 at 3, 7.5, or 30 mg/kg daily for 6 days starting 16 hours postinfection. LAMB administered at 7.5 mg/kg was used as a comparator arm. *F. solani* 95–2478 was chosen for these studies because it is among the most common causes of fusariosis; it demonstrated acceptable susceptibility to SF001 (MIC = 0.5 µg/mL using the 100% inhibition endpoint, Table 1); and it is the strain repeatedly used in mouse models of hematogenously disseminated infection (19–21). In two independent experiments (Fig. S1), neutropenic mice (*n* = 10 mice/group/experiment, for a total of 20 mice/group) infected with *F. solani* and treated with either SF001 at 7.5 or 30 mg/kg had a 21-day survival rate of 40% and 25%, respectively, which was comparable to the survival rate of mice treated with 7.5 mg/kg LAMB at 30% (*P* > 0.7 for SF001 at 7.5 or 30 mg/kg vs. LAMB). All three treatment regimens were significantly better than 0% survival for placebo-treated mice (*P* < 0.0001). Mice treated with SF001 at 3.0 mg/kg had a 21-day survival of 5% which was not statistically better than placebo mice (Fig. 1A). Consistent with the percent survival, SF001 at 7.5 or 30 mg/kg, or LAMB at 7.5 mg/kg, prolonged median survival to 10, 9, or 12.5 days, respectively, versus 6.5 days for placebo (*P* < 0.0001 for SF001 and *P* < 0.005 for LAMB), whereas SF001 at 3.0 mg/kg had a median survival of 7 days (Fig. 1B).

**Fig 2:**
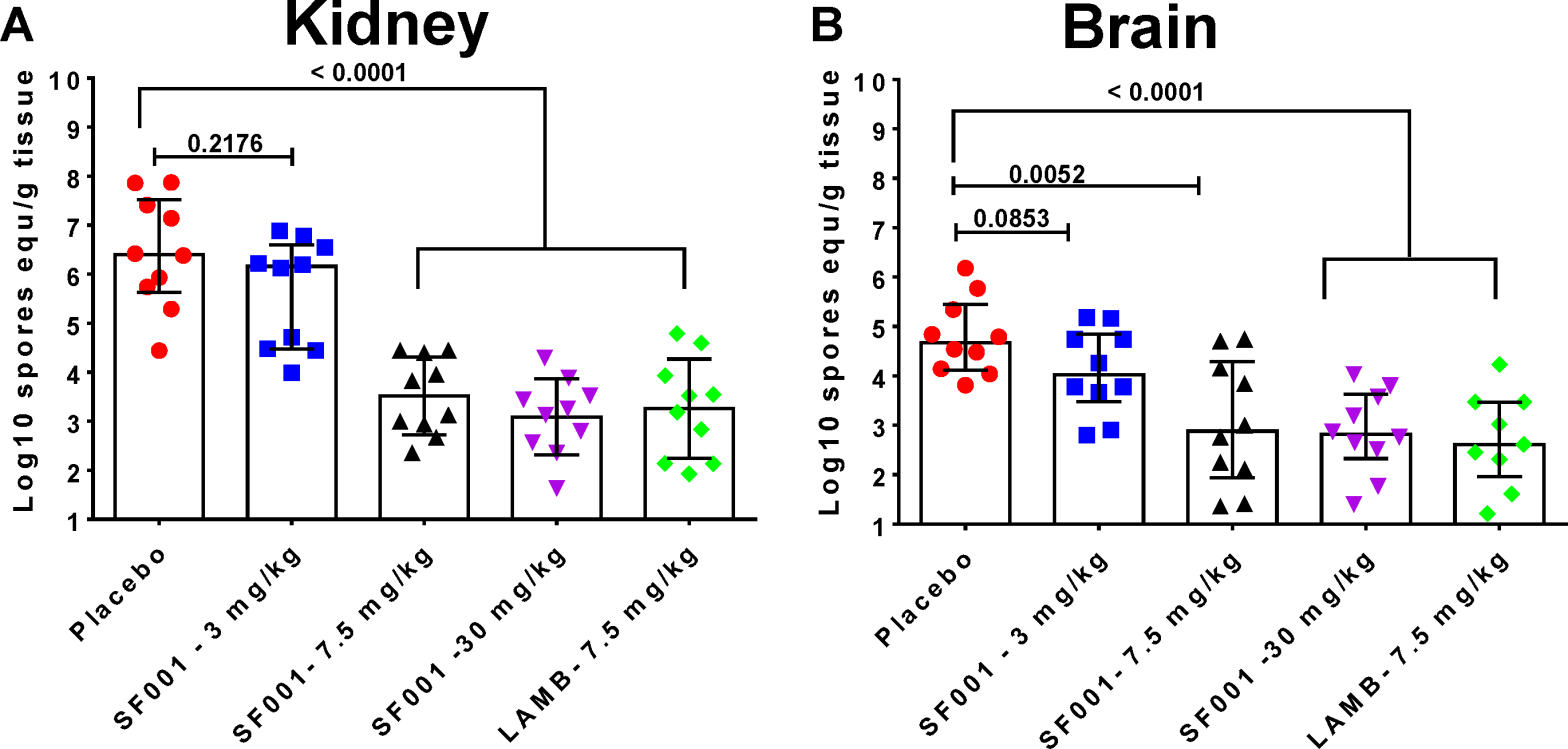
SF001 and LAMB lower kidney and brain fungal burden in immunosuppressed mice infected with *F. solani*. Mice (*n* = 10/group) intravenously infected with *F. solani* 95–2478 and treated daily with SF001 or LAMB were euthanized on day +4 postinfection ~6 hours after the last treatment. (**A**) In the kidneys, *P* < 0.0001 for SF001 7.5 and 30 mg/kg and for LAMB 7.5 mg/kg vs placebo. (**B**) In the brain, *P* < 0.005 for SF001 7.5 mg/kg, *P* < 0.0001 for SF001 30 mg/kg or LAMB 7.5 mg/kg vs placebo.

Because SF001 treatment improved survival of mice infected with *F. solani* over placebo treatment, the effects of SF001 on the tissue fungal burden in target organs of kidneys and brain were evaluated. Mice were infected and treated as described above and were humanely euthanized ~6 hours after the last treatment on day 4 post-inoculation. Kidneys and brains were then harvested and processed for tissue fungal burden and histopathology examination. Treatments with SF001 at 7.5 or 30 mg/kg resulted in ~3-log_10_ reduction in kidney fungal burden when compared with placebo (*P* < 0.0001). This reduction in kidney fungal burden was equivalent to the reduction demonstrated by LAMB (*P* < 0.0001) (Fig. 2A). The activity of SF001 or LAMB at the doses tested (Fig. 2B) was mirrored in the brain to a lesser extent, where a 2-log reduction in conidial equivalents (CEs) was observed (*P* < 0.005 for SF001 at 7.5 mg/kg vs placebo or *P* < 0.0001 for SF001 at 30 mg/kg or LAMB 7.5 mg/kg vs placebo).

**Fig 3:**
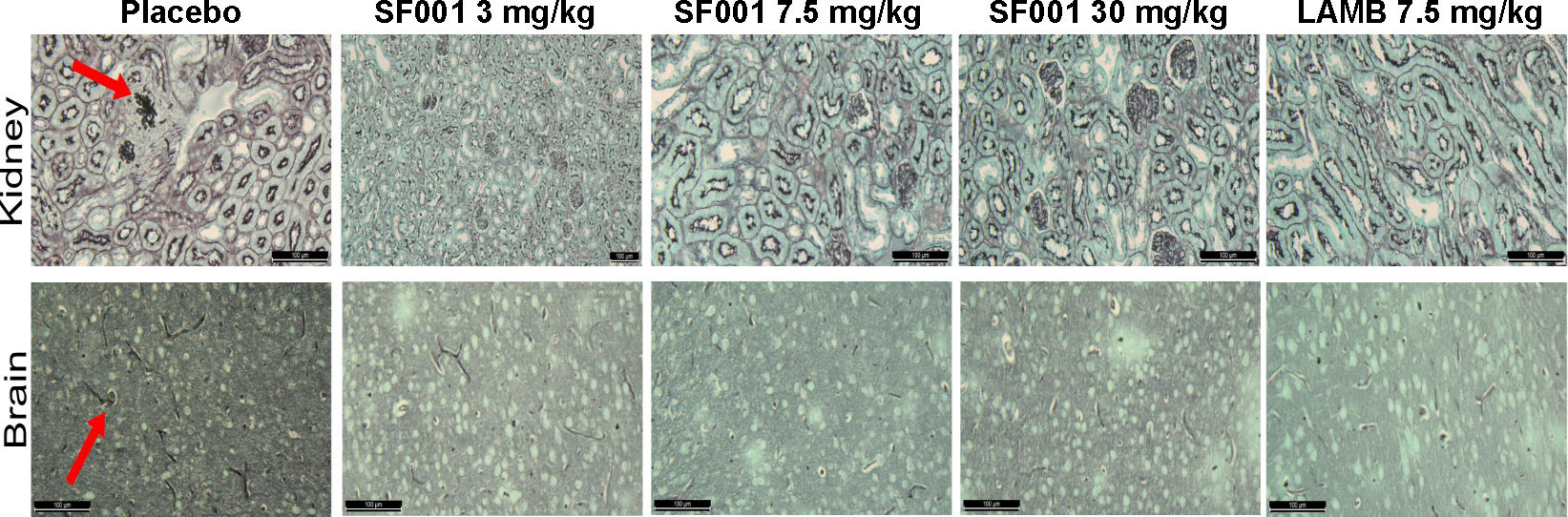
Histological examination of kidney and brain tissues harvested on day +4 from mice infected with *F. solani*. SF001 and LAMB demonstrated less hyphae in kidney and brain tissues vs placebo-treated mice. Arrows indicate fungal hyphae. The bar is 100 µm magnification.

To further correlate the findings of tissue fungal burden, histopathological examination was conducted on the same organs processed for the tissue fungal burden experiment. As expected, placebo-treated mice and, to a lesser extent, those treated with SF001 at 3 mg/kg, had abscesses filled with fungal hyphae in both the kidneys and brain, whereas mice treated with SF001 at 7.5 or 30 mg/kg or LAMB at 7.5 mg/kg had normal organ architecture with no signs of infection (Fig. 3). Collectively, these results demonstrate similar activity of SF001 at 7.5 and 30 mg/kg to the current standard of care of LAMB at a 7.5 mg/kg dose.

## DISCUSSION

Cases of invasive fusariosis have alarmingly been increasing among patients with hematologic malignancies. While AMB-based drugs and, in some cases, azole (e.g., voriconazole) therapy are used to treat these infections, the outcome is generally poor with high mortality rates (22). A contributing factor to the failure of therapy is the toxicity associated with AMB-based drugs, which limits drug dose escalation and reduces *in vitro* activity (i.e., higher MIC values), which leads to a poor therapeutic index. Novel drugs that enhance activity while reducing toxicity are needed to improve treatment outcomes.

SF001 is a novel, next-generation polyene with broad-spectrum activity and reduced systemic toxicity, particularly in the kidneys (15). When the MIC values of SF001 were compared with LAMB against seven clinical isolates of *F. solani* and six clinical isolates of *F. oxysporum,* enhanced *in vitro* activity of SF001 was found with MIC values (at 100% inhibition) ranging from 0.5 to 8 µg/mL for SF001 vs 1 to > 16 µg/mL for LAMB (with four *F*. *solani* and all six *F*. *oxysporum* isolates had LAMB MIC values of >16 µg/mL). SF001 also demonstrated *in vivo* efficacy in treating immunosuppressed mice from hematogenously disseminated fusariosis. This efficacy was manifested by the following: (1) enhanced overall survival of mice treated with doses >7.5 mg/kg; (2) prolonged median survival; (3) drastic reduction in tissue fungal burden of two target organs; and (4) improved histology of the target organs. Importantly, SF001 efficacy was comparable to that of LAMB in all the four tested criteria above. It is equally important to point out that SF001 was well tolerated in immunosuppressed mice, with no visual toxicity when administered a high dose of 30 mg/kg for six consecutive days, confirming previous reports of enhanced safety features of the drug (15). A recent study evaluating the *in vivo* pharmacodynamics (PK) of SF001 also showed that immunosuppressed mice tolerated doses of the drug up to 64 mg/kg (16).

A limitation of this study is the absence of *in vivo* testing of SF001 against other clinically relevant species of *Fusarium* (e.g., *F. oxysporum*). Also, it is not fully understood why the higher dose of 30 mg/kg of SF001 did not result in enhanced efficacy outcomes when compared with the 7.5 mg/kg dose (*P* = 0.54); however, equivalency was observed between LAMB and the 7.5 mg/kg and 30 mg/kg doses of SF001 (*P* > 0.7 for all comparisons). It is known that the single-dose PK of SF001 at both doses results in serum trough levels in excess of the MIC by several folds for at least 20 hours (16). Nevertheless, we did not observe any evidence of toxicity with the higher dose of 30 mg/kg, consistent with the lack of toxicity at 64 mg/kg reported in another study (16). Despite these limitations, these preclinical studies demonstrating SF001’s efficacy and low toxicity support continued investigation and development of SF001 as a novel polyene for the treatment of fusariosis.

## MATERIALS AND METHODS

### Isolates and culture conditions

*F. solani* 95–2478 is a blood isolate provided by P. Ferrieri (University of Minnesota). Other *F. solani* strains (FS1, FS2, FS3, FS4, FS5, and FS6) and *F. oxysporum* strains (FO1, FO2, FO3, FO4, FO5, and FO6) are clinical isolates obtained from the Fungus Testing Laboratory at the University of Texas Health Science Center at San Antonio. The organisms were grown on Sabouraud Dextrose Agar (SAB) for 4–7 days at 37°C. The macroconidia were collected in endotoxin-free PBS containing 0.01% Tween 80, washed with PBS, and then counted with a hemocytometer to prepare the final concentration.

### Susceptibility testing

*In vitro* susceptibility of SF001 (Elion Therapeutics, New York, NY, USA), amphotericin B powder (AMB, Sigma-Aldrich, Burlington, MA, USA), or LAMB (Gilead Sciences Inc., Foster City, CA, USA) against *F. solani* or *F. oxysporum* isolates was evaluated using the Clinical Laboratory and Standards Institute (CLSI) M38 broth microdilution method. Drugs were reconstituted to yield final working concentration following the manufacturer’s instructions. MIC values were read at 50% and 100% inhibition of growth compared with the growth control after 48 hours of incubation at 35°C. For each agent, the concentration tested ranged from 0.03 to 16 µg/mL.

### Immunosuppression

Male CD-1 mice (20–25 g from Envigo, Indianapolis, IN, USA) were used in this study. Mice were rendered neutropenic by administering cyclophosphamide (200 mg/kg, intraperitoneal injection) and cortisone acetate (500 mg/kg, subcutaneous injection) on days −2 and +3 relative to infection. This treatment regimen results in ~14 days of leukopenia with total white blood cell count dropping from ~130,000/cm^3^ to almost no detectable leukocytes as determined by Unopette System (Becton- Dickinson and Co.) (23). To prevent bacterial infection, 50 mg/L enrofloxacin (Bayer, Leverkusen, Germany) was added to the drinking water on day −3 and then switched to daily ceftazidime treatment (5 mg/mouse, subcutaneous injection) starting on the day of infection (day 0) to day 13 (24).

### Infection and treatment

On day 0, mice were infected through tail vein injection with a targeted inoculum of 8.0 × 10^2^/conidia per mouse. Treatment with placebo (diluent control 5% DW), SF001 (3, 7.5, or 30 mg/kg once daily [QD]), or LAMB (7.5 mg/kg QD; Gilead, Foster City, CA, USA) began 16 hours post infection and continued for 6 days through the intravenous route. The primary and secondary endpoints were time to moribundity (survival) and tissue fungal burden in the kidneys and brain (primary and secondary target organs), measured by CEs per gram of tissue by qPCR (25). We also conducted histological examination of kidney and brain sections taken from mice representative of all groups of treatment and stained with Grocott’s methenamine silver stain for microscopic examination (*n* = 2/group). Control groups of uninfected, neutropenic mice were also included in the survival studies. Animal studies were approved by the IACUC at the Lundquist Institute at Harbor-UCLA Medical Center, according to the NIH guidelines for animal housing and care.

### Statistical analysis

For survival studies, based on the vast experience with animal models, it was expected that 10 mice/group would provide at least 80% power to test the hazard ratio of 0.2 or less, with a level of significance *P* = 0.025 using the Cox proportional hazard model (one-sided test), assuming 100% and 50% mortality in the test and control group, respectively. For the tissue pathogen burden, 10 mice/group would provide approximately 90% statistical power to detect the effect size of 2.5 or 2.5 SD difference in CFU (expressed as log) content using a two-sided two-sample *t*-test with an α of 0.05, assuming the standard deviation of the test group is twice that of the control group. For all comparisons, mean ± SD, median (interquartile range), and 95% CI were computed, and *P* values of <0.05 were considered significant. All data analyses were conducted using GraphPad Prism 6.

## SUPPLEMENTAL MATERIAL

Fig. S1 aac.01802-24-s0001.docx Survival curves for two independent experiments assessing the efficacy of SF001 or LAMB in treating immunosuppressed mice with hematogenously disseminated fusariosis due to *F. solani*.

ASM does not own the copyrights to Supplemental Material that may be linked to, or accessed through, an article. The authors have granted ASM a non-exclusive, world-wide license to publish the Supplemental Material files. Please contact the corresponding author directly for reuse.
