# Supplementary material for: Efficacy assessments of SF001, a next-generation polyene antifungal, in a neutropenic mouse model of invasive fusariosis
Source: Antimicrob Agents Chemother. 2025 Apr 1;69(5):e01802-24. doi: 10.1128/aac.01802-24 (PMC12057364; doi:10.1128/aac.01802-24)
Supplement: Fig. S1 — Survival curves for two independent experiments assessing the efficacy of SF001 or LAMB in treating immunosuppressed mice with hematogenously disseminated fusariosis due to F. solani. [file aac.01802-24-s0001.docx]

**Supplemental Figure. Survival curves for two independent experiments assessing the efficacy of SF001 or LAMB in treating immunosuppressed mice with hematogenously disseminated fusariosis due to *F. solani*.** Mice (n= 10/group per experiment) were infected by tail vein and treated with either placebo, SF001(3, 7.5, or 30 mg/kg), or LAMB (7.5 mg/kg) for 6 consecutive days. Inoculum for experiment 1 (EXP-1) was 8.6 x 10^2^ conidia, and for experiment 2 (EXP-2) was 9.5 x 10^2^ condia.
